# Formate addition enhanced hydrogen production by *Thermococcus paralvinellae* when grown on brewery wastewater

**DOI:** 10.3389/fmicb.2025.1560780

**Published:** 2025-03-18

**Authors:** Harita Sistu, James F. Holden

**Affiliations:** Department of Microbiology, University of Massachusetts, Amherst, MA, United States

**Keywords:** biohydrogen, hyperthermophile, *Thermococcus*, formate, hydrogenase

## Abstract

The hyperthermophilic archaeon *Thermococcus paralvinellae* produces H_2_ when grown on carbohydrates or protein with increased H_2_ production when cultures are grown on formate. This study examined the use of brewery wastewater as a feedstock for H_2_ production, the addition of formate to enhance H_2_ production, and the activities of hydrogenases and formate hydrogenlyase under varying growth conditions as markers of performance. *T. paralvinellae* was grown at 80°C on maltose only (a model brewery wastewater), formate only, and maltose plus formate media as well as brewery wastewater with and without the addition of formate. Growth rates were higher on formate only medium than on maltose only and brewery wastewater only media. H_2_ yield per cell was higher in all media containing formate relative to those without formate. Hydrogenase and formate hydrogenlyase specific activities were not affected by the presence of formate and were largely consistent across all growth conditions. Growth rates were consistent in media containing 0.05 to 2.5% (wt/vol) maltose only, but total H_2_ production doubled from medium containing 0.05% maltose to 0.5% maltose and remained unchanged at higher maltose concentrations. Cells grown in a 2 L N_2_ flushed batch bioreactor at 80°C on brewery wastewater with and without formate showed no difference in growth rates but the amount of H_2_ in the headspace was six times higher when formate was present. However, the amount of H_2_ produced by cells grown on brewery wastewater plus formate peaked in mid-logarithmic growth phase and then decreased to amounts produced by cells without formate addition by late logarithmic growth phase. When the bioreactor was run as a chemostat, the addition of formate to brewery wastewater led to a 12-fold increase in the amount of H_2_ present in the headspace that was sustained over time relative to growth without formate addition. Therefore, *T. paralvinellae* grows on brewery wastewater as its sole source of organic carbon and produces biohydrogen at a steady rate in a pilot-scale bioreactor, and H_2_ production is enhanced by formate addition.

## 1 Introduction

According to a projection by the U.S. Energy Information Administration in its *International Energy Outlook 2023* (U.S. Energy Information Administration, 2023), global energy consumption will increase by 34% and the corresponding energy-related carbon emissions will increase by 15% by the year 2050. All projections anticipate an increase in renewable energy use. Consequently, interest in H_2_ as a source of renewable energy has increased (Bockris, 1972; Jain, 2009; Pivovar et al., 2018). However, most industrial H_2_ today is produced by hydrolyzing water using energy from fossil fuels with high carbon emissions (Marchant, 2021). Alternatively, green H_2_ is produced from renewable sources such as wind, solar, or biomass and could play a key role toward a carbon-neutral future.

Microbial biohydrogen production also shows promise (Show et al., 2011; Zhou et al., 2022). Specifically, biohydrogen production via dark fermentation has advantages such as high H_2_ production rates and the use of substrates such as organic-rich waste and wastewater. Increased reaction temperatures can further boost biohydrogen production rates, improve mixing, and reduce contamination by pathogens and H_2_-consuming methanogens (Chou et al., 2008; Verhaart et al., 2010; Gupta et al., 2016). Consequently, interest in high temperature H_2_-producing microorganisms is increasing. Among hyperthermophilic archaea, *Thermococcales* produce H_2_ in the absence of zero-valence sulfur (S^0^) (Zillig and Reysenbach, 2015). They use a ferredoxin-dependent membrane-bound hydrogenase (Mbh) for H_2_ production that is coupled with H^+^/Na^+^ translocation for ATP synthesis (Sapra et al., 2003). Various *Thermococcus* species were used to produce H_2_ using waste substrates such as keratin-rich animal waste (Bálint et al., 2005), waste milk and spent grain from breweries (Hensley et al., 2016), chitin (Aslam et al., 2017), lipid-extracted microalgae (Chen et al., 2020), and potato peel waste (Lee et al., 2023). However, due to thermodynamic limitations, H_2_ can reach inhibitory concentrations (Verhaart et al., 2010).

To ameliorate H_2_ inhibition, some *Thermococcus* species can reduce CO_2_ using H_2_ and produce formate using a membrane-bound formate hydrogenlyase (Fhl) (Topçuoğlu et al., 2018; Le Guellec et al., 2021). The *mbh* and *fhl* operons in *Thermococcus* are often adjoining on the same DNA strand (Holden and Sistu, 2023), and each operon is regulated by the SurR transcription factor that is activated by the absence of S^0^ (Lipscomb et al., 2009, 2017; Yang et al., 2010) suggesting that the operons are linked and co-regulated. The Fhl reaction is reversible; therefore, *Thermococcus* species with Fhl and a membrane-bound formate transporter can also oxidize formate to H_2_ and CO_2_ coupled with H^+^/Na^+^ translocation and ATP synthesis on the membrane (Kim et al., 2010; Bae et al., 2012, 2015; Lim et al., 2014). Topçuoğlu et al. (2018) reported 81-fold higher H_2_ production when *Thermococcus paralvinellae* was grown on formate versus maltose. Therefore, the ability to produce H_2_ from organic compounds and formate makes *Thermococcus* a good candidate for organic waste-to-H_2_ conversion. However, the combined effect of formate and maltose (or any other organic compound) on H_2_ production in *Thermococcus* is not known.

**Figure 1:**
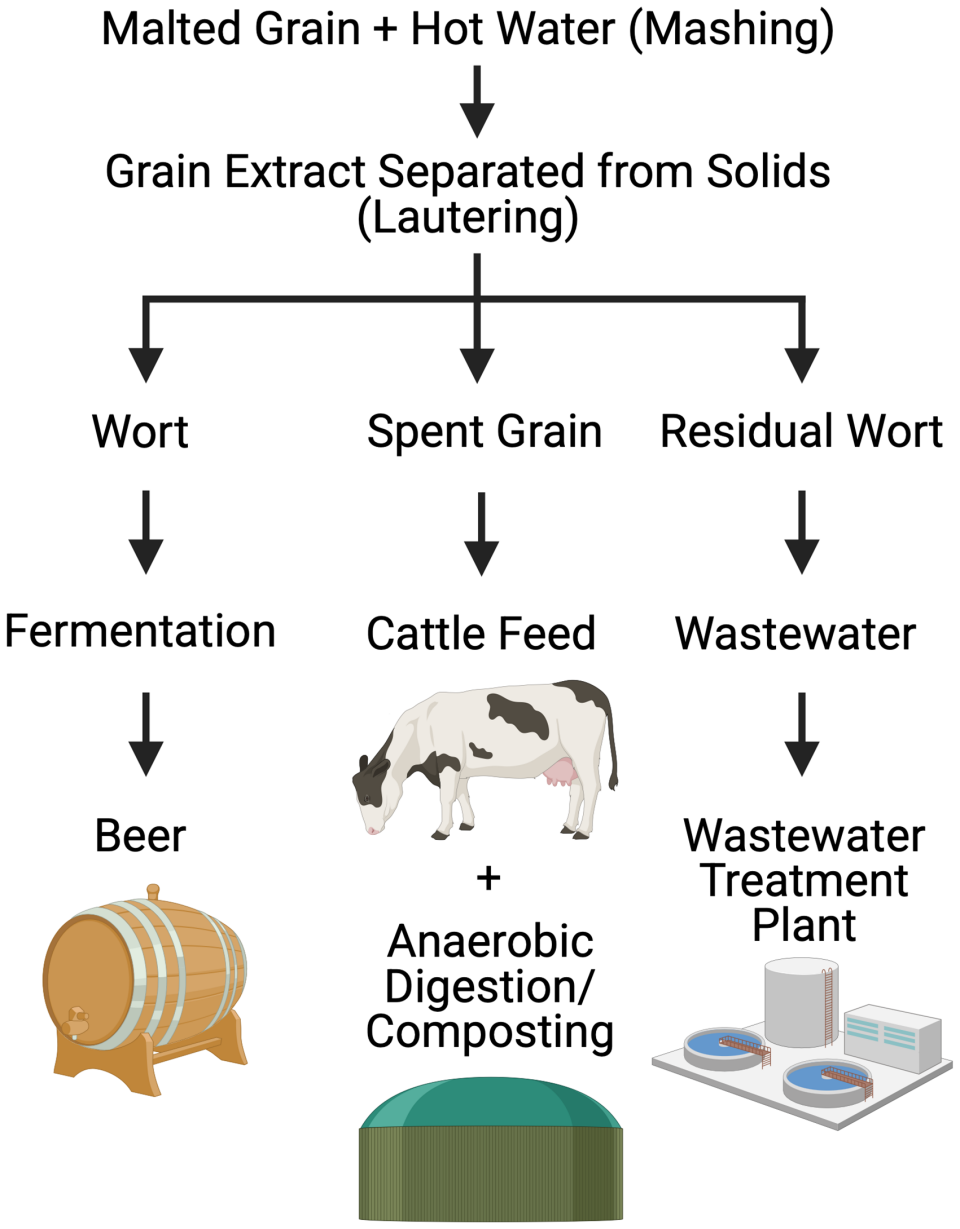
Simplified flow-diagram depicting pre-fermentation steps in the beer-making process, highlighting waste generation and treatment. Created in BioRender by H. Sistu https://BioRender.com/n02b647.

In this study, brewery wastewater, specifically residual wort (Figure 1), was identified as a model waste stream. Brewing is a water-intensive process, with 3–10 L of organic-rich wastewater discharged per L of beer produced (Ashraf et al., 2021). The brewing process starts with malted grain soaked in hot water to extract sugars from grain in a process called mashing, followed by lautering to separate the sugar water from the grain. The resulting liquid is called wort, a solution of extracted grains that is approximately 90% carbohydrates (He et al., 2014). Maltose and maltotriose are the most abundant sugars found in wort followed by sucrose, fructose, and glucose. Residual wort is a particle-rich pre-fermentation waste stream that is separated from wort prior to the addition of yeast and hops for wort fermentation. Its high organic load makes it a challenge to remediate, especially in urban and suburban settings. In Massachusetts, largescale brewery wastewater discharge into landfills, wastewater treatment plants, or local waterbodies without pretreatment is prohibited due to eutrophication risks.

We examined the effect of adding formate separately to brewery wastewater and maltose medium (a model brewery wastewater) on growth rates and H_2_ production by *T. paralvinellae*. Specific activities of hydrogenase and Fhl were measured to determine if they covary with growth condition and as biochemical markers to optimize waste-to-H_2_ conversion. Lastly, optimum organic waste substrate concentration was determined, and a pilot-scale bioreactor was run to determine growth rates and H_2_ production using brewery wastewater with and without formate as a substrate for waste-to-H_2_ conversion. The results show that *T. paralvinellae* grows on brewery wastewater as its sole source of organic carbon and produces biohydrogen at a steady rate in a pilot-scale bioreactor, that the addition of formate to organic waste significantly increased H_2_ production, and that growth rates and the activities of hydrogenases and Fhl were largely stable with variations in growth media.

## 2 Materials and methods

### 2.1 Organism used and growth conditions

*Thermococcus paralvinellae* ES1 (DSM 27261) (Hensley et al., 2014) was obtained from the Deutsche Sammlung für Mikroorganismen und Zelkulturen (Braunschweig, Germany). Its growth medium was based on DSM 282 medium (Jones et al., 1983) and consisted of the following (per liter): 30 g of NaCl, 4.1 g of MgCl_2_∙6H_2_O, 3.4 g of MgSO_4_∙7H_2_O, 0.33 g of KCl, 0.25 g of NH_4_Cl, 0.14 g of CaCl_2_∙2H_2_O, 0.14 g of KHPO_4_, 1 mM potassium phosphate buffer (pH 6.8), 10 mL of DSM 141 trace minerals solution, 10 mL of DSM vitamins solution, 50 μL of 0.5% (wt/vol) resazurin, 0.1 g of yeast extract (Difco, vitamin fortified), 1 mL of 0.2% (wt/vol) (NH_4_)_2_Fe(SO_4_)_2_ and 0.2% (wt/vol) (NH_4_)_2_Ni(SO_4_)_2_ combined solution, and 0.1 mL of 10 mM Na_2_WO_4_∙2H_2_O. Five combinations of carbon and energy sources were added separately to the base medium (per liter): (A) 5 g of maltose monohydrate as a model of brewery wastewater, (B) 1 g of sodium formate, (C) 5 g maltose plus 1 g sodium formate, (D) 25% (vol/vol) brewery wastewater, and (E) 25% brewery wastewater plus 1 g sodium formate, unless otherwise stated. The brewery wastewater consisted of residual wort (Figure 1) and was obtained from White Lion Brewery (Springfield, Massachusetts). Suspended solids in the brewery wastewater were removed by centrifugation at 10,000 × g for 20 min followed by filtering through a 0.22 μm MCE membrane filter (Millipore). This step was repeated. All media were pH balanced to 6.80 ± 0.05 and reduced with 0.025% (wt/vol) each of cysteine-HCl and Na_2_S∙9H_2_O before inoculation.

*T. paralvinellae* was grown at 80°C in 1.6 L of growth medium in 2 L sealed bottles with stirring (150–180 rpm) on maltose, formate, maltose plus formate, brewery wastewater, and brewery wastewater plus formate for growth rate, H_2_ yield (amount per cell), and enzyme activity measurements. It was also grown on different concentrations of maltose (per liter, 0.5 g, 5 g, 10 g, and 25 g) to determine the optimal concentration of organic carbon for H_2_ production. Growth rates and H_2_ yields were determined for each condition. Prior to inoculation, the bottles were sealed with butyl rubber stoppers and the headspace was flushed and filled with 1 atm N_2_. Each growth medium was inoculated with 50 mL of cells in logarithmic growth phase. The cells were adapted to each growth medium prior to inoculation by transferring them at least three times on the medium. Cells were grown in at least triplicate for each growth condition. For enzyme activity measurements, the cells were harvested in late logarithmic growth phase by centrifugation at 10,000 × g, resuspended in degassed and N_2_ flushed 50 mM MOPS buffer (pH 7.5) that was reduced with 2 mM sodium dithionite, and stored under N_2_ at-20°C until further use.

*T. paralvinellae* was also grown at 80°C in a 2 L bioreactor with stirring (150–180 rpm) in 1.6 L of base medium on 10% (vol/vol) brewery wastewater only or 10% brewery wastewater plus 1 g/L of sodium formate, each without added yeast extract. The media were sparged with pure N_2_ at a rate of 120 mL/min. Each growth medium was inoculated with 50 mL of cells in logarithmic growth phase. For batch cultivation, cells were incubated in triplicate for 96 h in each growth medium. For chemostatic growth, cells were incubated in batch phase until they reached mid-logarithmic growth phase and then switched to chemostat phase by pumping reduced growth medium into the reactor from a 12 L reservoir sparged with N_2_ and heated to 80°C (Supplementary Figure S1). Simultaneously, spent medium was pumped out of the reactor at the same rate using a dual-channel peristaltic pump. The dilution rate for the chemostat was set to 5 mL/min based on previous batch cultivation growth rates. Each chemostat was run for at least three volume exchanges. At various time points, samples of the growth medium and headspace were removed for cell counts and H_2_ measurements for both batch cultivation and chemostatic growth.

### 2.2 Measurements of growth rate, H_2_, formate, and chemical oxygen demand

To determine the specific growth rate of the cells, the cell concentration in the bottles and bioreactor was measured at various time points during logarithmic growth phase using a Petroff-Hausser counting chamber and phase-contrast light microscopy. The specific growth rate (*k*) was determined by plotting the cell concentration against time and fitting an exponential curve to the data. The amount of H_2_ in the headspace of the bottles and bioreactor was measured at various time points using a gas chromatograph equipped with a 6′ Hayesep D column (Restek), argon as the carrier gas, and a thermal conductivity detector. Spent growth medium was sampled at the end of growth for formate measurements, centrifuged at 10,000 × g for 10 min, filtered through a 0.22 μm MCE membrane filter (Millipore), and stored at -20°C. Formate was measured enzymatically and colorimetrically using an MAK059 Formate Assay Kit (Sigma-Aldrich) according to the manufacturer’s protocol. Soluble chemical oxygen demand (sCOD) was measured using a Hach COD kit (salt/seawater TNT816 – High Range (70–1,000 mg/L)) according to manufacturer’s protocol. For batch cultivation in the bioreactor, samples for sCOD were removed every 12 h starting at time of inoculation. Suspended solids were removed from the samples by centrifugation at 10,000 × g for 10 min and filtration through a 0.22 μm MCE membrane filter. The samples were diluted prior to testing so that the concentration was within detection range. The sCOD of the brewery wastewater was measured directly using the Hach COD digestion vials High Range (50–1,500 mg/L) kit.

### 2.3 Enzyme activity measurements

Hydrogenase, formate hydrogenlyase, and formate dehydrogenase activities were measured from cells grown in 2 L sealed bottles. All enzyme activities were performed at 80°C using degassed and N_2_ flushed 50 mM MOPS buffer (pH 7.5) containing 20 mM NaCl, 2 mM MgCl_2_, and 2 mM sodium dithionite (Lipscomb et al., 2014). Hydrogenase and formate dehydrogenase activities were measured after the cell pellets were thawed and sonicated in an anoxic chamber to generate whole cell extract. There was no formate hydrogenlyase activity in whole cell extracts suggesting that the enzyme complex was disrupted by sonication. Therefore, formate hydrogenlyase activity was measured using washed and concentrated intact cells after centrifugation but prior to freezing. H_2_-evolving hydrogenase and formate hydrogenlyase activities were determined by measuring the amount of H_2_ produced at various time points in rubber stopper-sealed serum vials (10 mL) that were flushed with N_2_. The H_2_ was measured using a gas chromatograph as described above. For the H_2_-evolution hydrogenase assay, the enzyme buffer contained 3 mM methyl viologen reduced with 30 mM sodium dithionite as the electron donor and the reaction was initiated by the addition of whole cell extract (Sapra et al., 2000). The rate of H_2_ production was determined by plotting the amount of H_2_ produced against time and fitting a linear regression line to the data. For the formate hydrogenlyase assay, the enzyme buffer contained 25 mM sodium formate as the electron donor (Lipscomb et al., 2014) and the reaction was initiated by the addition of intact washed and concentrated cells. The rate of H_2_ production was determined by plotting the amount of H_2_ produced against time and fitting a linear regression line to the data.

H_2_-oxidation hydrogenase activity was determined by spectrophotometrically measuring at 600 nm the reduction of 3 mM benzyl viologen [*ε* = 7,400/(M∙cm)] contained in rubber stopper-sealed glass cuvettes under a H_2_ headspace (Bryant and Adams, 1989; Ma et al., 2000). Activity was initiated by the addition of whole cell extract. Formate dehydrogenase activity was determined by spectrophotometrically measuring at 600 nm the reduction of 3 mM benzyl viologen by whole-cell extract contained in rubber stopper-sealed glass cuvettes under a N_2_ headspace (Ma et al., 1995). Sodium formate (25 mM) was used as electron donor and activity was initiated by the addition of formate. All enzyme activities were expressed as units where 1 U is equal to 1 μmol of H_2_ or formate produced or consumed per min. Activities were normalized by protein concentration of the whole cell extracts for each sample. The protein concentrations of the whole cell extract were determined using the DC Protein Assay kit (Bio-Rad). Bovine serum albumin was used as the standard.

### 2.4 Statistical analysis

Illustrations (unless stated otherwise), analysis of variance (ANOVA), and Tukey’s Honest Significant Difference tests were performed using R Statistical Software (v4.4.2; R Core Team, 2024) with the R Packages tidyvere (v2.0.0; Wickham et al., 2019) and rstatix (v0.7.2; Kassambara, 2023). Compared values were considered significantly different when *p* < 0.05.

## 3 Results and discussion

### 3.1 Growth rates, H_2_ production, and enzyme activities on defined media and brewery wastewater

**Figure 2:**
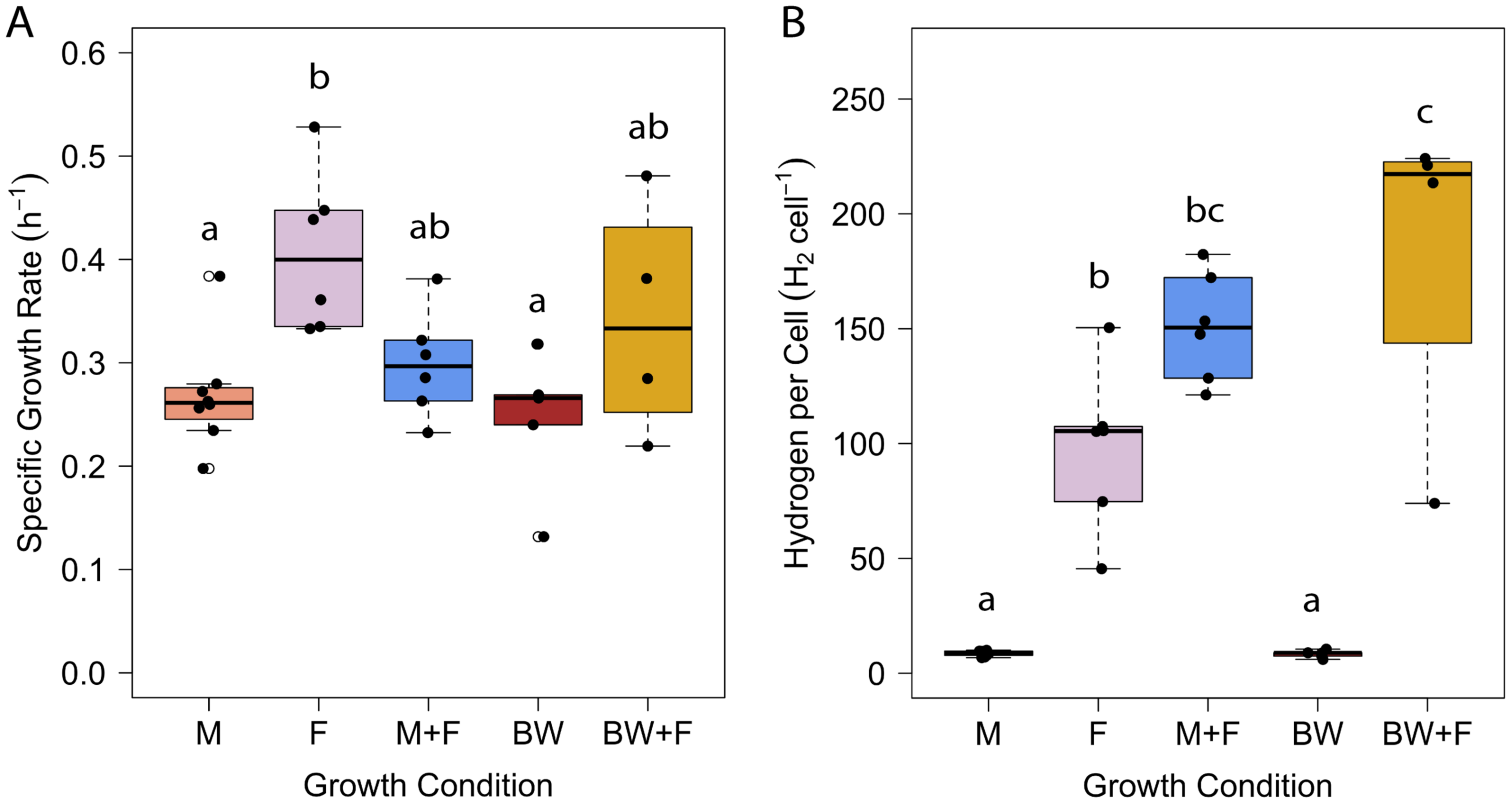
Specific growth rate **(A)** and H_2_ yield per cell **(B)** of *T. paralvinellae* when grown on maltose only (M), formate only (F), maltose + formate (M + F), brewery wastewater only (BW), and brewery wastewater + formate (BW + F). Each box represents the interquartile range, with the top and bottom of the box depicting the first quartile and third quartile, respectively. The vertical bar depicts the median, the dots depict single data points, and error bars depict the minimum and maximum intensities. The statistical relevance of the data is *p* < 0.05, and the lowercase letters indicate statistically similar groups.

Brewery wastewater, specifically residual wort (Figure 1), was identified as a model waste stream based on 26 customer discovery interviews as part of the National Science Foundation’s I-Corps™ program at University of Massachusetts Amherst (see Supplemental material). *T. paralvinellae* was grown separately in stirred, sealed bottles on brewery wastewater and maltose medium with and without formate added and on formate only. The specific growth rate was higher on formate only than on maltose only and brewery wastewater only (Figure 2A). The cell doubling time was 102 min on formate only (*k* = 0.41/h ± 0.07/h [±95% confidence interval]) compared to 155 min (*k* = 0.27/h ± 0.04/h) and 170 min (*k* = 0.25/h ± 0.08/h) for maltose only and brewery wastewater only, respectively (Supplementary Table S1). There was no significant difference in specific growth rates between maltose plus formate and brewery wastewater plus formate and the other conditions. *T. paralvinellae* doubled every 139 min (*k* = 0.30/h ± 0.05/h) on maltose plus formate and every 122 min (*k* = 0.34/h ± 0.16/h) on brewery wastewater plus formate. When cells were grown on maltose only and brewery wastewater only, the H_2_ yields per cell (Figure 2B) were comparable at 9 ± 1 fmoles/cell (±95% confidence interval) and 8 ± 2 fmoles/cell, respectively (Supplementary Table S1). The H_2_ yield per cell on formate only was 98 ± 34 fmoles/cell and was significantly higher than the yields for maltose only and brewery wastewater only (Figure 2B). The H_2_ yield per cell on brewery wastewater plus formate was 183 ± 101 fmol/cell and was significantly higher than the yields for maltose only, formate only, and brewery wastewater only (Figure 2B). The H_2_ yield per cell on maltose plus formate was 151 ± 23 fmol/cell and was significantly higher than the yields on maltose only and brewery wastewater only (Figure 2B).

It was previously reported that *T. paralvinellae* grew faster on maltose than on formate in a chemostat but that the rate of H_2_ production was higher on formate than on maltose (Topçuoğlu et al., 2018). In this study, the growth rate was higher in bottles on formate only than on maltose only (Figure 2A). However, it was previously unknown what the effect would be on growth rates and H_2_ production by combining formate with maltose or other organic substrates. The specific growth rate data herein showed that growth in stirred bottles remained unchanged across eight of the 10 pairwise media comparisons and that the combination of formate with another carbon source significantly increased the amount of H_2_ produced per cell relative to the same media without formate. Therefore, enhanced H_2_ production by *T. paralvinellae* is due primarily to the presence of formate and is not repressed by the addition of maltose or brewery wastewater to formate. The results suggest that biomass production is dependent primarily on the maltose, yeast extract, or brewery waste organic compounds present in the medium and is generally not affected by formate utilization. The formate added is most likely not assimilated into cell biomass but instead is oxidized by formate hydrogenlyase to increase H_2_ production and H^+^/Na^+^ translocation outside of the cell and thus increase ATP synthesis for the cell (Topçuoğlu et al., 2018).

The genome of *T. paralvinellae* ES1 (Jung et al., 2014) encodes for seven hydrogenases, namely two membrane-bound ferredoxin-dependent hydrogenases (*mbh*), a soluble NAD(P)^+^-dependent hydrogenase (*sh*), two membrane-bound formate hydrogenlyases (*fhl*), a membrane-bound carbon monoxide dehydrogenase (*codh*), and a soluble H_2_-oxidizing regulatory protein (*frh*) linked to SurR regulation (Jung et al., 2020). *T. paralvinellae* previously showed H_2_-evolving hydrogenase activity in its insoluble (e.g., membrane) protein fraction and H_2_-oxidizing hydrogenase activity in its soluble (e.g., cytoplasmic) protein fraction in cells grown on maltose and tryptone without S^0^ (Hensley et al., 2016). *T. paralvinellae* produced formate when grown separately on maltose and tryptone (Hensley et al., 2016; Topçuoğlu et al., 2018) and H_2_ when grown on formate (Topçuoğlu et al., 2018). It expressed the genes for all seven of its hydrogenases when grown separately on maltose, tryptone, and formate, and *fhl* expression was highest when cells were grown on formate (Topçuoğlu et al., 2018). However, Fhl enzyme activity from *Thermococcus* has only been measured when Fhl from *Thermococcus onnurineus* was produced heterologously in *Pyrococcus furiosus* (Lipscomb et al., 2014). Native Fhl activity had not been measured in *Thermococcus*, nor had its activity been compared with the activities of its other hydrogenases under varying growth conditions.

**Figure 3:**
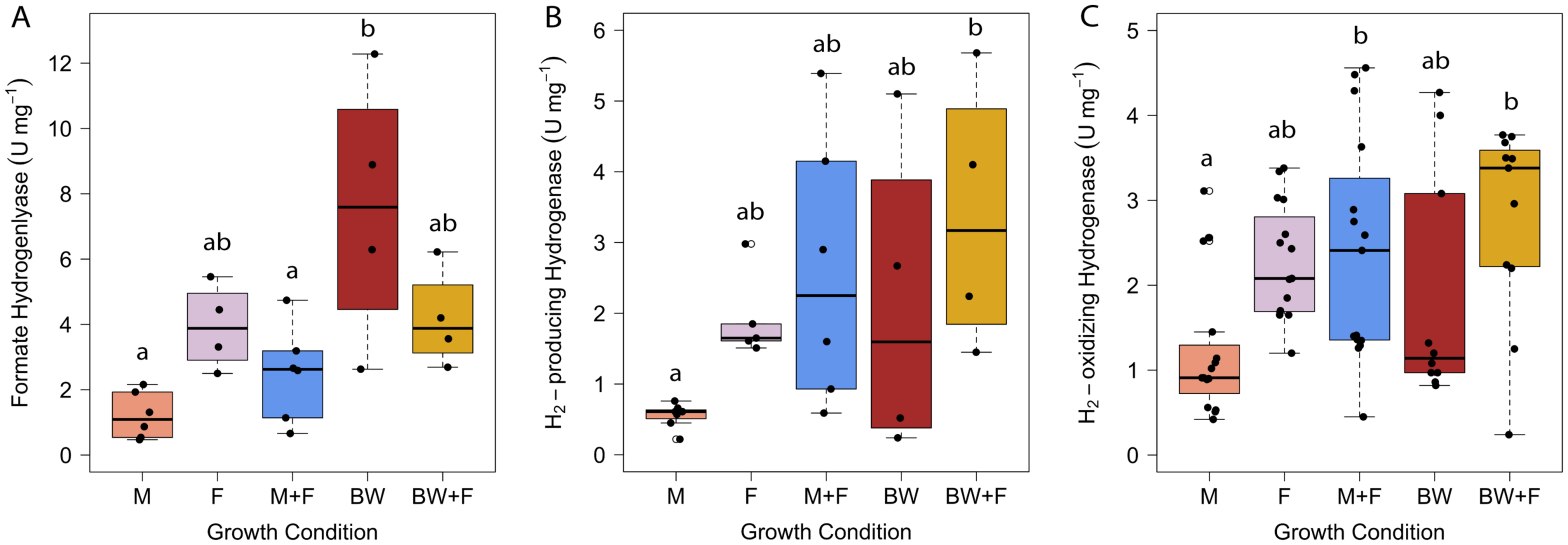
Specific activities of formate hydrogenlyase **(A)**, H_2_-producing hydrogenase **(B)**, and H_2_-oxidizing hydrogenase **(C)** in *T. paralvinellae* when grown on maltose only (M), formate only (F), maltose + formate (M + F), brewery wastewater only (BW), and brewery wastewater + formate (BW + F). Each box represents the interquartile range, with the top and bottom of the box depicting the first quartile and third quartile, respectively. The vertical bar depicts the median, the dots depict single data points, and error bars depict the minimum and maximum intensities. The statistical relevance of the data is *p* < 0.05, and the lowercase letters indicate statistically similar groups.

Hydrogenase and formate hydrogenlyase activities were measured in *T. paralvinellae* and remained generally consistent across all five growth conditions (Figure 3). Fhl activity was measured in washed and concentrated intact cells but was not detectable in whole cell extracts suggesting that the membrane-bound 14-subunit enzyme complex was disrupted and inactivated by sonication. Western blot analysis of membrane and cytoplasmic protein fractions for the catalytic subunit of Fhl from *Thermococcus litoralis* similarly showed that most of the catalytic subunit was in the cytoplasmic fraction suggesting it dissociated from the rest of the enzyme complex in the membrane (Takács et al., 2008). Unlike previous *fhl* transcript abundances that were higher in *T. paralvinellae* grown on formate relative to maltose (Topçuoğlu et al., 2018), the presence of formate in this study did not increase Fhl enzyme activity in *T. paralvinellae* relative to media without formate (Figure 3A). Fhl activity was low in cells grown on maltose and maltose-plus-formate relative to those grown on brewery wastewater only. Formate (0.19 mM ± 0.04 mM) was produced when *T. paralvinellae* was grown on maltose only, most likely to ameliorate H_2_ stress (Topçuoğlu et al., 2018), which may explain why Fhl activity was the same as in formate only grown cells. Formate dehydrogenase activity was negligible for cells from all growth conditions except for brewery wastewater only where Fdh activity was 38 ± 30 U/mg (Supplementary Figure S2; Supplementary Table S2). For all other conditions, specific Fdh activities were very low and ranged from 0.04 ± 0.01 U/mg to 1.7 ± 1.9 U/mg. Therefore, Fhl and Fdh may be more abundant in cells grown on brewery wastewater due to H_2_ inhibition and oxidation to form formate to ameliorate H_2_ inhibition.

H_2_-producing hydrogenase activity was lower in maltose only grown cells relative to brewery wastewater-plus-formate grown cells (Figure 3B). Similarly, H_2_-oxidizing hydrogenase activity was lower in maltose only grown cells relative to brewery wastewater-plus-formate and maltose-plus-formate grown cells (Figure 3C). In *P. furiosus*, which is phylogenetically closely related to *Thermococcus* (Zillig and Reysenbach, 2015), methyl viologen-dependent H_2_-producing hydrogenase activity is generally representative of the membrane-bound ferredoxin-dependent Mbh hydrogenase (Sapra et al., 2000) and benzyl viologen-dependent H_2_-oxidizing hydrogenase activity representative of the cytoplasmic NAD(P)^+^-dependent Sh hydrogenase (van Haaster et al., 2008). However, both Mbh and Sh can catalyze both reactions. Therefore, each hydrogenase activity reported herein likely represents the pooled Mbh and Sh hydrogenase activities of *T. paralvinellae*.

Formate hydrogenlyase and hydrogenase activities were generally uniform across all growth conditions except that they were all lowest in maltose only grown cells. These results suggest that formate and H_2_ metabolizing enzyme abundance in *T. paralvinellae* remain relatively unchanged in maltose medium and brewery wastewater with and without added formate and on formate only. It also suggests that *fhl* and *mbh* are regulated by SurR and the absence of S^0^ only, and that the presence of formate does not up-or down-regulate either of these operons independently. The increase in H_2_ production with added formate was likely due to an increase in substrate availability rather than an increase in Fhl abundance. Therefore, *T. paralvinellae* is primed physiologically to increase H_2_ production rapidly when provided with formate. The optimum concentration of formate for H_2_ production by *T. paralvinellae* during growth on organic substrates would need to be determined. The growth, H_2_ production, and enzyme activity results suggest that *T. paralvinellae* is a promising candidate for H_2_ production using organic waste substrates and that H_2_ production can be enhanced by adding formate during growth. Like formate, carbon monoxide was also shown to stimulate H_2_ production in *Thermococcus onnurineus* (Bae et al., 2012; Lee et al., 2022). Therefore, carbon monoxide oxidation coupled with organic waste remediation would be an alternative procedure for H_2_ production. A summary of formate hydrogenlyase, hydrogenase, and formate dehydrogenase activities is found in Supplementary Table S2.

### 3.2 Optimal substrate concentration

**Figure 4:**
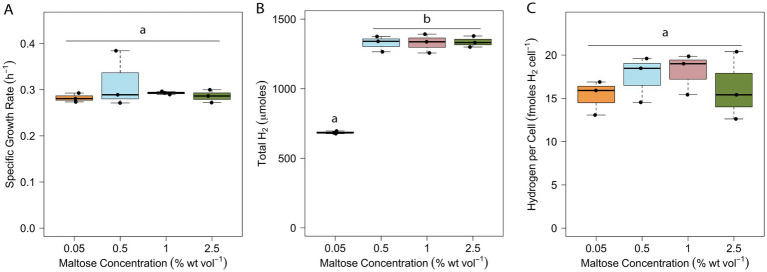
Specific growth rate **(A)**, total headspace H_2_ **(B)**, and H_2_ yield per cell **(C)** of *T. paralvinellae* when grown on various concentrations of maltose. Each box represents the interquartile range, with the top and bottom of the box depicting the first quartile and third quartile, respectively. The vertical bar depicts the median, the dots depict single data points, and error bars depict the minimum and maximum intensities. The statistical relevance of the data is *p* < 0.05, and the lowercase letters indicate statistically similar groups.

The optimal substrate concentration for brewery wastewater-to-H_2_ conversion by *T. paralvinellae* was determined by measuring its growth rate and total H_2_ production on 0.05 to 2.5% maltose. The growth rate did not change with an increase in substrate concentration (Figure 4A). The average doubling times ranged from 132 min (*k* = 0.32/h ± 0.12/h) to 147 min (*k* = 0.28/h ± 0.02/h) (Supplementary Table S3). The total H_2_ produced doubled from 685 μmoles ±20 μmoles on 0.05% maltose to 1,327 μmoles ±114 μmoles on 0.5% maltose (*p* < 0.001) and remained at the latter amount of total H_2_ at higher maltose concentrations (Figure 4B; Supplementary Table S3). The H_2_ yield per cell was unchanged at all maltose concentrations (Figure 4C) and ranged from 15 ± 4 fmoles/cell with 0.05% maltose to 18 ± 5 fmoles/cell with 1% maltose (Supplementary Table S3). Stoichiometrically, up to eight moles of H_2_ are produced from one mole of maltose (Verhees et al., 2003). The highest amount of H_2_ produced per mole of maltose available was 0.5 moles of H_2_ per mole of maltose in the 0.05% maltose bottles suggesting that relatively little maltose is needed to support the growth of *T. paralvinellae*. While growth rates and H_2_ yield per cell remained unchanged with increase in maltose concentration, the increase and plateau of total H_2_ produced at 0.5% maltose meant that this was the optimum organic substrate concentration for operating a pilot-scale waste-to-H_2_ bioreactor using *T. paralvinellae*.

### 3.3 Pilot-scale conversion of brewery wastewater-to-H_2_

Optimum concentration of brewery wastewater for the pilot-scale bioreactor was determined based on chemical oxygen demand (COD), which is a measure of the amount of oxygen required to chemically oxidize organic matter in a solution. The soluble COD of 0.5% maltose only medium (i.e., the minimum maltose concentration for maximum H_2_ production) was 5,706 ± 315 mg COD/L while that of brewery wastewater was 60,967 ± 6,034 mg COD/L (Supplementary Figure S3; Supplementary Table S4). Therefore, brewery wastewater was diluted tenfold with artificial seawater for operation of the pilot-scale bioreactor.

**Figure 5:**
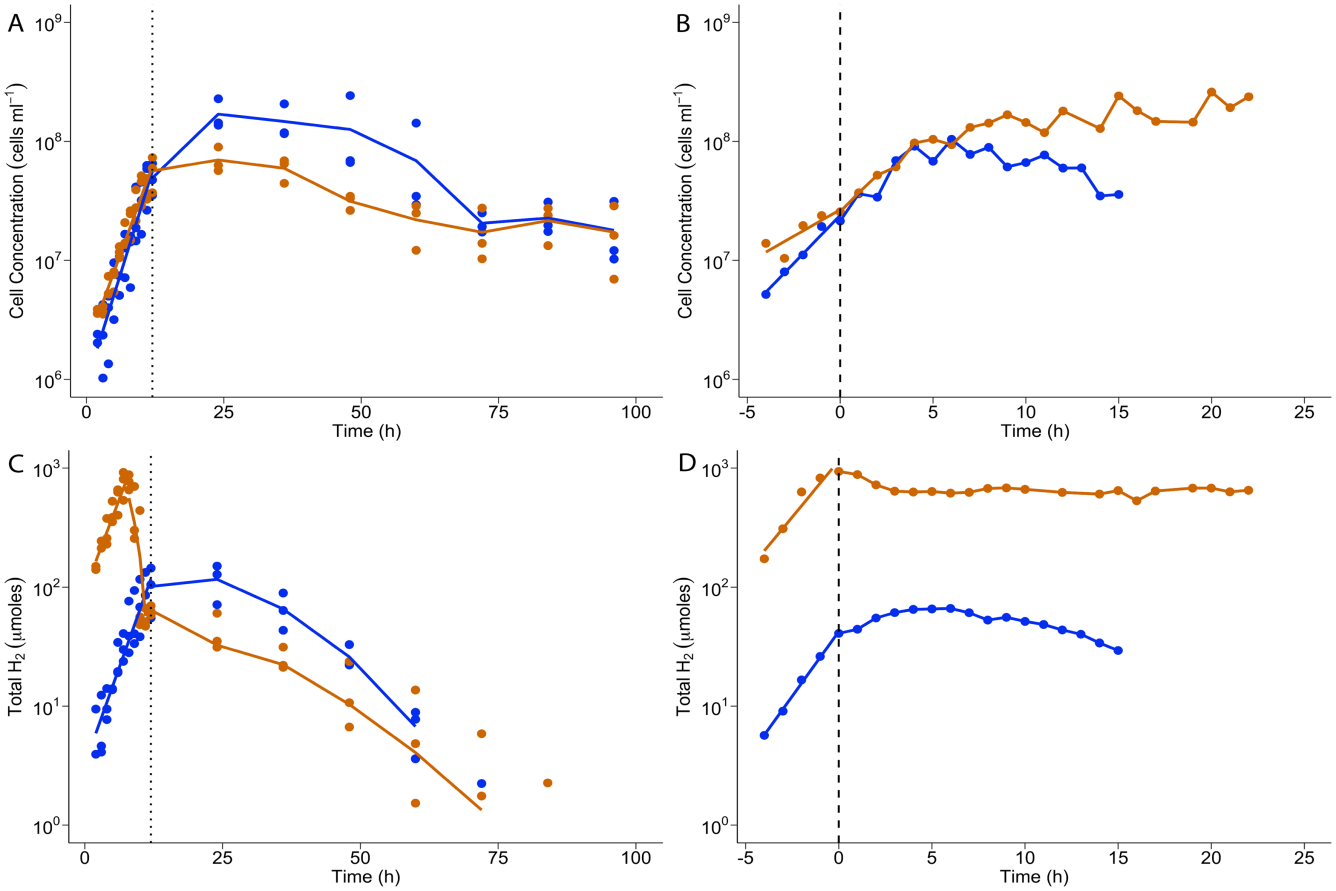
*T. paralvinellae* cell concentrations **(A,B)** and total headspace H_2_ **(C,D)** when cells were grown by batch cultivation **(A,C)** and in a chemostat **(B,D)**. Cells were grown on brewery wastewater only (blue) or brewery wastewater plus formate (orange). The dotted lines **(A,C)** indicate the end of logarithmic growth phase during batch cultivation; the dashed lines **(B,D)**, the start of growth in chemostatic phase following logarithmic growth in batch cultivation.

When grown in batch cultivation, *T. paralvinellae* grew at the same rate on brewery wastewater with and without added formate. Cells doubled every 117 min (*k* = 0.36/h ± 0.06/h) on brewery wastewater only and every 141 min (*k* = 0.29/h ± 0.04/h) on brewery wastewater plus formate (Figure 5A; Supplementary Table S5). Stationary phase cell concentrations were similar for both conditions and remained unchanged until 48 h when concentrations decreased. Maximum cell concentration for both conditions was observed at 24 h and was 1.7 × 10^8^ cells/ml ± 1.0 × 10^8^ cells/ml for brewery wastewater only and 7.0 × 10^7^ cells/ml ± 2.5 × 10^7^ cells/ml for brewery wastewater plus formate (Figure 5A; Supplementary Table S6). The amount of H_2_ in the headspace increased during logarithmic growth phase when *T. paralvinellae* was grown on brewery wastewater only, and the maximum amount was 116 μmoles ±83 μmoles at 24 h (Figure 5C; Supplementary Table S7). The amount of H_2_ in the headspace was significantly higher when cells were grown on brewery wastewater plus formate with the maximum amount being 741 μmoles ±366 μmoles at 7 h, approximately six-times higher than the maximum headspace H_2_ when grown on brewery wastewater only (Figure 5C). However, the amount of H_2_ decreased from mid-logarithmic growth to late logarithmic growth to levels comparable to growth on brewery wastewater only. H_2_ yield per cell also showed similar trends, dropping after 5 h when *T. paralvinellae* was grown on brewery wastewater plus formate suggesting H_2_ inhibition (Supplementary Figure S4A; Supplementary Table S8). Soluble COD (sCOD) remained unchanged with time during growth on brewery wastewater only and reduced by 6% from initial values after 48 h when grown on brewery wastewater plus formate (Supplementary Figure S5; Supplementary Table S9). However, initial sCOD in brewery wastewater plus formate was also 6% higher than brewery wastewater only, most likely due to the addition of the formate that contributes to COD (Aristova et al., 2003). Therefore, the COD data suggest that all the formate was consumed in the brewery wastewater plus formate medium, which may explain the decrease in H_2_ amount during logarithmic growth.

To determine if formate-enhanced H_2_ production could be sustained, *T. paralvinellae* was grown in a chemostat to achieve steady state growth and H_2_ production using brewery wastewater with and without formate. Growth rates and cell concentrations were comparable between both conditions (Figure 5B; Supplementary Table S10). Steady-state cell concentrations in the chemostat for growth on brewery wastewater only were 6.9 × 10^7^ cells/ml ± 0.2 × 10^7^ cells/ml, while for brewery wastewater plus formate they were 1.7 × 10^8^ cells/ml ± 4.8 × 10^6^ cells/ml. H_2_ production was significantly higher during growth on brewery wastewater plus formate relative to growth on brewery wastewater only, with high levels of H_2_ production being sustained in the chemostat (Figure 5D; Supplementary Table S10). The H_2_ production rate was 5,110 μmoles/h ± 143 μmoles/h for growth on brewery wastewater plus formate, approximately 12-times higher than that of brewery wastewater only where the H_2_ production rate was 415 μmoles/h ± 57 μmoles/h. H_2_ yield per cell for growth on brewery wastewater plus formate dropped slightly after switching to chemostatic growth due to an increase in cell concentration but remained significantly higher than the H_2_ yield per cell on brewery wastewater only (Supplementary Figure S4B; Supplementary Table S10). Therefore, *T. paralvinellae* grew consistently and comparably on brewery wastewater with and without formate in a pilot-scale bioreactor. Furthermore, enhanced and sustained H_2_ production occurred when cells were grown in a chemostat with the addition of formate.

## 4 Conclusion

Brewery wastewater is an environmental issue for many breweries due to the high organic load of residual wort and the risks of overwhelming the treatment capabilities of wastewater treatment plants and eutrophication of water bodies. There is also a need for biohydrogen to ameliorate our global energy consumption needs. This study demonstrated that brewery wastewater could be used as a feedstock for thermophilic microbial growth with concomitant biohydrogen production, and that H_2_ production increased significantly by formate addition. Hydrogenases and formate hydrogenlyase were present and growth rates were consistent under all conditions tested indicating that H_2_ production was only dependent on formate availability. *Thermococcus* is a marine organism; therefore, sea salts must be added to brewery wastewater or other organic waste feedstocks for growth and H_2_ production. Sodium formate is a safe and easy to handle salt that is comparable in low cost to the other salts added for growth. The increase in COD was minor and following incubation decreased to the COD level found in brewery wastewater without formate. This suggests the formate is largely converted to H_2_ and CO_2_ and does not increase the COD level of the waste following incubation. Thus, the organic compounds in the waste are used for biomass production and formate supplementation is amenable to enhanced H_2_ production. The organic waste can be removed as cell mass and formate addition can make the process more cost effective by utilizing the enhanced biohydrogen.

The process was run in a 2 L bioreactor suggesting that it is amenable to operation using various carbohydrate-and protein-rich waste streams in a medium-sized (e.g., <1,000 L) reactor with rapid turnover rates and a relatively small spatial footprint. The amount of waste remediated into biomass was low suggesting that future designs should consider cell removal and recirculation of the waste to enhance its conversion. Furthermore, the putative acetate produced in the spent medium could be used as a feedstock for thermophilic methanogenesis in a downstream process. A two-step fermentation process using the H_2_-producing thermophilic bacterium *Caldicellulosiruptor saccharolyticus* followed by mesophilic methanogenesis was used to generate H_2_ and methane from garden and food waste with the goal of optimizing bioenergy production (Abreu et al., 2019). Whether formate-enhanced H_2_ production by *T. paralvinellae* can be a feasible solution to produce bioenergy will depend on energy yield and scalability. A life cycle assessment of this process is needed to compare energy cost for operating a high-temperature H_2_ production bioreactor with the energy potential of the H_2_ produced to determine feasibility of the process, and further studies are needed to effectively harvest H_2_ for use as energy.

## Data Availability

The original contributions presented in the study are included in the article/Supplementary material, further inquiries can be directed to the corresponding author.
