# Supplementary material for: Formate addition enhanced hydrogen production by Thermococcus paralvinellae when grown on brewery wastewater
Source: Front Microbiol. 2025 Mar 18;16:1560780. doi: 10.3389/fmicb.2025.1560780 (PMC11959275; doi:10.3389/fmicb.2025.1560780)
Supplement: Supplementary file 1 [file Data_Sheet_1.PDF]

Supplemental Material

Formate addition enhanced hydrogen production by *Thermococcus paralvinellae* when grown on brewery wastewater

Harita Sistu[1], James F. Holden[1*]
[1]Department of Microbiology, University of Massachusetts, Amherst, Massachusetts, USA

***Correspondence:**
James F. Holden
jholden@umass.edu

1. Supplemental text

NSF Innovation-Corps (I-Corps™) at the University of Massachusetts Amherst

26 customer discovery interviews were conducted as part of the University of Massachusetts Amherst I-Corps™ training program in the 2020-2021 cohort which consisted of three stages: Warm-Up, Jump-Start, and Rev-Up. All interviews were conducted over the telephone or by video. The goal was to identify existing problems and solutions for organic waste management in various industries. An initial business model canvas outlining the hypothesis by defining customers and their respective value propositions (that is, the value that a technology or service can provide for the customer) in the Jump-Start stage and 10 interviews were conducted to test the hypothesis. Professionals from university dining and sustainability services, wastewater treatment plants (WWTP), breweries, farms, and waste management companies were interviewed as customers to determine market needs of a waste-to-H_2 treatment process. A business model canvas was generated with customers including university dining services, wastewater treatment plants, breweries, and dairy farmers, and the respective value propositions being treating university-generated food waste, resolving the Massachusetts Department of Environmental Protection (MassDEP) commercial food material disposal ban, and managing waste milk. The MassDEP has issued a food material disposal ban prohibiting facilities from disposing more than half a ton of commercial organic/food waste per week. We found that universities have been exploring solutions to divert food waste from landfills. Existing solutions focused on composting. However, they were limited by the capacity of composting facilities compared to the large amount of food waste generated by universities, leading them to explore anaerobic digestion as an alternative. We interviewed a member from a farming community and found their biggest issue to be treatment of manure. We also interviewed a professional in the craft brewing industry and from a WWTP and learned that breweries had multiple waste streams that needed managing.

The hypothesis and business model canvas were updated upon concluding the Jump-Start interviews to focus on waste streams from breweries and their effect on WWTPs. Following Jump-Start, 16 professionals from breweries and WWTPs were interviewed in the Rev-Up stage. In the business model canvas for Rev-Up, breweries and WWTPs were the customers and treatment of brewery waste was the value proposition. Breweries' largest waste stream was spent grain. The grain is highly nutritious, so for craft breweries near farming communities, local farmers used the spent grain as cattle feed. It benefited both brewery and farmer since breweries need not pay for waste disposal and farmers receive free animal feed. Larger breweries and those located in cities partnered

with anaerobic digestion and composting facilities to treat spent grain. Liquid waste (wastewater) from different steps in the brewing process was flushed down the drain and sent to the WWTP. These steps included draining of residual wort, from residual yeast and hops mixture, and the wash down from rinsing fermenters. The burden that this wastewater put on WWTPs depended largely on the size and location of the brewery. When connected to city WWTPs, wastewater from most small- to mid-size breweries got diluted, presenting no need for pre-treatment, while large-scale breweries or those located in smaller towns had to pre-treat the wastewater or pay a surcharge for disposing organic-rich wastewater. Some breweries stored wastewater in tanks and drained them according to a timetable coordinated with the WWTP. Pre-treatment processes ranged from pH neutralization to anaerobic digestion depending on the needs of breweries and the respective WWTPs. A few anaerobic digestion companies that were serving breweries were identified, but their capacities were limited. Because of the organic-rich nature of brewery wastewater and the limited treatment technologies available on the market, brewery wastewater was determined to be an appropriate test waste stream to develop our pilot-scale hyperthermophilic waste-to-H_2 bioremediation process.

2. Supplemental figures and tables
2.1 Supplemental figures

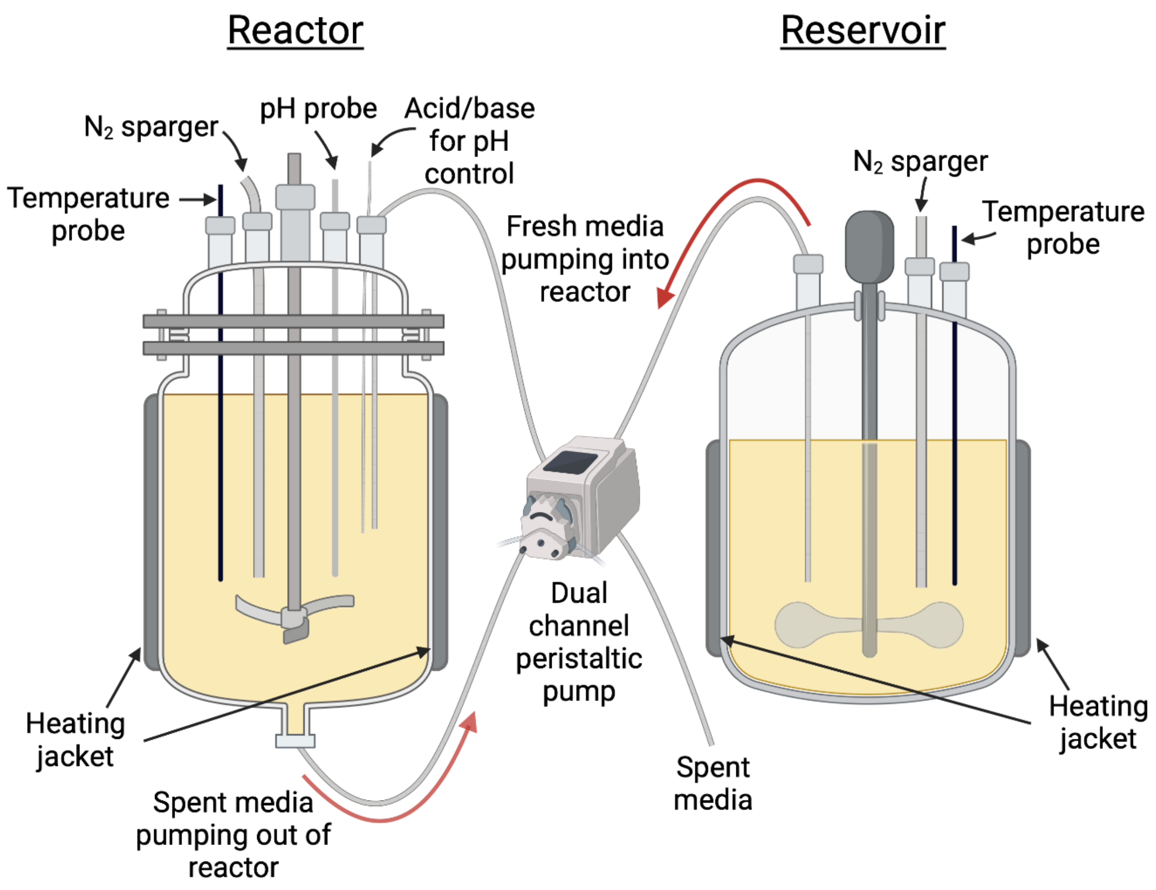

Figure S1. Schematic of the chemostat used for the incubation of brewery wastewater with and without the addition of sodium formate. Cells were grown in the anaerobic bioreactor that was heated to 80°C and sparged with N_2. Sterile growth medium in the reservoir was also maintained at 80°C and sparged with N_2. Sterile growth medium was pumped from the reservoir into the bioreactor while spent growth medium was pumped out of the reactor at the same rate using a dual channel peristaltic pump. The headspace and liquid of the bioreactor were sampled for H_2 and cell counts, respectively, throughout incubation. Created in BioRender. Sistu, H. (2025) https://BioRender.com/e36k796

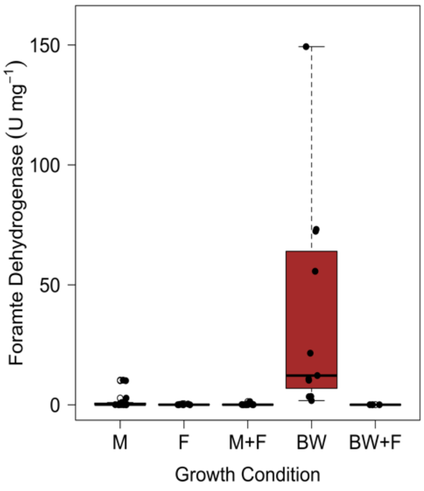

Figure S2. Formate dehydrogenase specific activities in *T. paralvinellae* when grown in a stirred sealed bottle on maltose only (M), formate only (F), maltose plus formate (M+F), brewery wastewater only (BW), brewery wastewater plus formate (BW+F). Each box represents the interquartile range, with the top and bottom of the box depicting the first quartile and third quartile, respectively. The vertical bar depicts the median, the dots depict single data points, and error bars depict the minimum and maximum intensities. The statistical relevance of the data is $p < 0.05$.

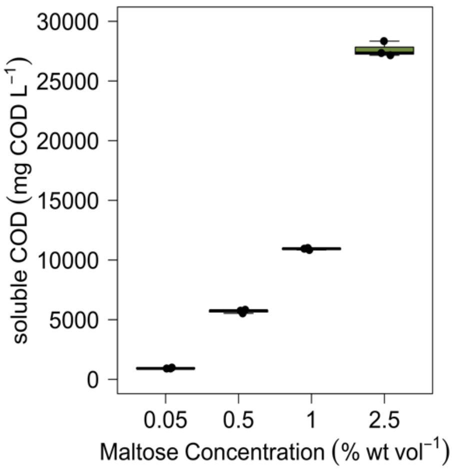

Figure S3. Initial soluble COD of maltose-containing media ranging from 0.05% to 2.5% maltose. Each box represents the interquartile range, with the top and bottom of the box depicting the first quartile and third quartile, respectively. The vertical bar depicts the median, the dots single data points, and error bars depict the minimum and maximum intensities.

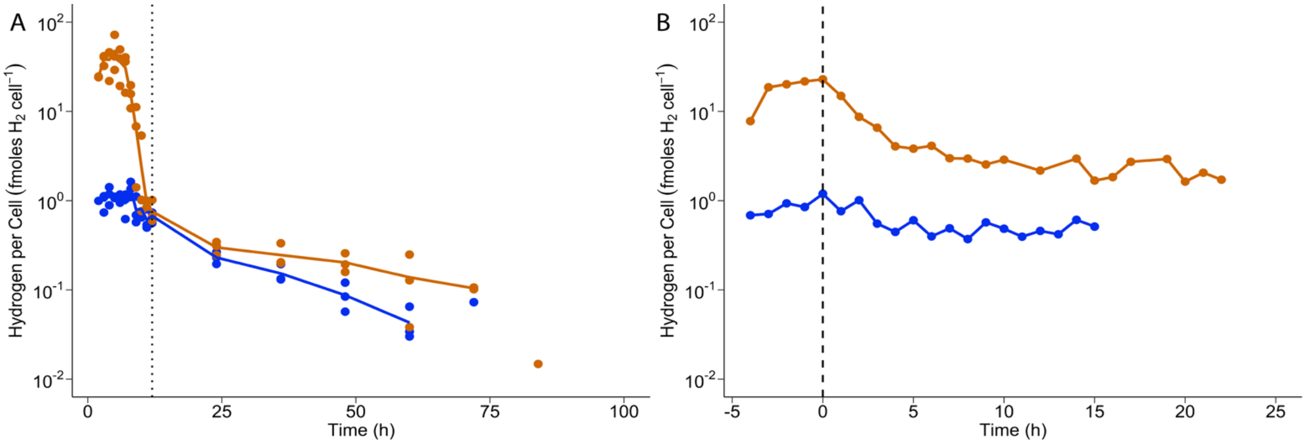

Figure S4. Hydrogen yield per cell for *T. paralvinellae* when grown on brewery wastewater only (●) or brewery wastewater plus formate (●) during batch cultivation (A) and in a chemostat (B). The dotted line (A) indicates the end of logarithmic growth phase during batch cultivation; the dashed line (B), the start of chemostatic growth following logarithmic growth in batch cultivation.

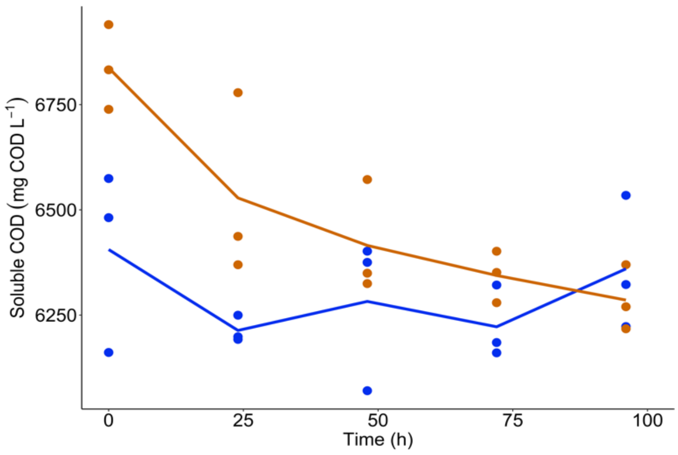

Figure S5. Soluble COD of spent growth media after *T. paralvinellae* was grown on brewery wastewater only (●) and brewery wastewater plus formate (●) during batch cultivation

2.2 Supplemental tables

Table S1. Specific growth rate (h^{-1}) and H_2 yield per cell (fmol/cell) for *T. paralvinellae* when grown on maltose only (M), formate only (F), maltose plus formate (M+F), brewery wastewater only (BW), and brewery wastewater plus formate (BW+F)

Expt.	Specific growth rate (h^{-1})					H_2 per cell (fmol/cell)				
	M	F	M+F	BW	BW+F	M	F	M+F	BW	BW+F
1	0.235	0.333	0.322	0.132	0.285	9.54	105.25	128.48	6.03	224.10
2	0.263	0.335	0.232	0.240	0.481	7.80	107.44	153.34	10.50	73.97
3	0.260	0.448	0.381	0.269	0.382	9.59	74.72	172.25	7.47	213.49
4	0.280	0.439	0.308	0.318	0.219	6.78	45.50	121.19	8.88	221.12
5	0.272	0.361	0.262	0.266		7.85	150.44	147.60	8.91	
6	0.198	0.528	0.286			7.10	105.61	182.33		
7	0.256					8.60				
8	0.384					9.63				
9						10.00				
Mean	0.268	0.407	0.299	0.245	0.342	8.54	98.16	150.87	8.36	183.17

Table S2. Specific activities (U/mg total protein) of formate hydrogenlyase, H_2-producing hydrogenase, H_2-oxidizing hydrogenase, and formate dehydrogenase in *T. paralvinellae* when grown on maltose only (M), formate only (F), maltose plus formate (M+F), brewery wastewater only (BW), and brewery wastewater plus formate (BW+F)

Expt.	Formate hydrogenlyase (U/mg)					H_2-producing hydrogenase (U/mg)					H_2-oxidizing hydrogenase (U/mg)					Formate dehydrogenase (U/mg)				
	M	F	M+F	BW	BW+F	M	F	M+F	BW	BW+F	M	F	M+F	BW	BW+F	M	F	M+F	BW	BW+F
1	0.47	4.45	0.66	12.28	4.20	0.45	2.98	0.59	5.10	4.1	0.90	2.50	1.26	4.27	0.24	0.05	0.43	0.13	72.4	0.05
2	0.87	5.46	1.14	6.29	6.22	0.61	1.85	5.39	0.24	5.68	0.91	2.43	1.35	3.08	1.25	0.05	0.34	0.12	149.3	0.07
3	2.16	2.50	4.74	2.63	3.56	0.61	1.61	4.15	0.52	2.24	0.89	2.07	1.4	4.00	3.68	0.04	0.02	0.05	10.8	0.04
4	0.54	3.31	3.19	8.89	2.69	0.22	1.51	2.90	2.67	1.45	1.09	3.34	0.45	0.97	3.50	0.49	0.02	0.06	12.2	0.03
5	1.93		2.59			0.66	1.65	1.60			1.45	3.38	4.56	0.97	3.49	0.24	0.04	0.50	10.3	0.03
6	1.31		2.66			0.57		0.93			0.53	3.03	4.48	1.08	2.24	0.22	0.05	0.03	3.40	0.04
7						0.76					0.52	1.68	1.36	0.82	2.20	0.08	0.13	0.03	3.48	
8											1.14	1.65	1.29	0.86	2.96	0.08	0.18	0.03	1.76	
9											1.02	1.20	1.41	1.20	3.77	2.80	0.05	1.34	21.5	
10											0.91	3.01	4.29	1.32	3.75	0.96		0.03	73.1	
11											0.51	2.60	2.41		3.38	0.07		0.09	55.7	
12											0.56	1.85	3.63			0.17		0.05		
13											3.11	1.65	2.89			0.02				
14											2.56	1.70	2.75			10.3				
15											2.52	2.08	2.59			10.1				
Mean	1.21	3.93	2.50	7.52	4.17	0.55	1.92	2.59	2.13	3.37	1.23	2.28	2.41	1.86	2.77	1.70	0.14	0.20	37.62	0.04

Table S3. Specific growth rate (h^{-1}), total headspace H_2 (µmoles), H_2 yield per cell (fmol/cell), and H_2 per maltose (mol/mol) for *T. paralvinellae* when grown on various concentrations of maltose

Expt.	Specific growth rate (h^{-1})				Total headspace H_2 (µmol)				H_2 per cell (fmol/cell)				H_2 per maltose (mol/mol)			
	0.05%	0.5%	1.0%	2.5%	0.05%	0.5%	1.0%	2.5%	0.05%	0.5%	1.0%	2.5%	0.05%	0.5%	1.0%	2.5%
1	0.274	0.289	0.296	0.272	676.92	1340.42	1392.17	1378.93	13.07	19.60	15.42	15.41	0.490	0.097	0.050	0.020
2	0.272	0.384	0.293	0.286	682.60	1375.34	1337.07	1331.59	15.91	14.54	19.00	20.39	0.490	0.099	0.048	0.019
3	0.273	0.271	0.289	0.300	695.92	1265.12	1257.03	1299.49	16.89	18.47	19.86	12.63	0.500	0.091	0.045	0.019
Mean	0.282	0.315	0.293	0.286	685.15	1326.96	1328.76	1336.67	15.29	17.54	18.09	16.14	0.493	0.096	0.048	0.019

Table S4. Soluble chemical oxygen demand (COD) of uninoculated maltose media and undiluted brewery wastewater

Experiment	Soluble COD (mg COD/L)				
	0.05% maltose	0.5% maltose	1.0% maltose	2.5% maltose	Brewery wastewater
1	914	5532	10948	28341	63950
2	911	5755	11005	27158	58000
3	985	5831	10841	27342	60950
Mean	937	5706	10932	27613	60967

Table S5. Specific growth rate (h^{-1}) of *T. paralvinellae* when grown in batch cultivation on brewery wastewater only (BW) and brewery wastewater plus formate (BW+F)

Expt.	Growth rate (h^{-1})	
	BW	BW+F
1	0.331	0.313
2	0.387	0.297
3	0.347	0.272
Mean	0.355	0.294

Tables S6. Cell concentrations (× 10^7/ml) of *T. paralvinellae* at various time points when cells were grown in batch cultivation on brewery wastewater only and brewery wastewater plus formate

Time	Brewery wastewater only				Brewery wastewater plus formate			
(h)	1	2	3	Mean	1	2	3	Mean
2	0.20	-	0.24	0.22	0.39	0.36	-	0.37
3	0.23	0.10	0.43	0.25	0.41	0.37	0.35	0.38
4	0.50	0.14	0.4	0.35	0.74	0.52	0.53	0.59
5	0.79	0.32	0.96	0.69	0.76	0.80	0.55	0.70
6	1.07	0.51	0.74	0.77	1.31	1.05	1.17	1.18
7	1.27	0.72	1.66	1.22	2.08	1.40	1.36	1.61
8	1.60	0.59	1.43	1.21	2.61	2.46	1.48	2.18
9	1.88	1.46	4.14	2.49	3.92	2.77	2.15	2.95
10	3.20	1.66	4.54	3.13	5.14	4.60	2.87	4.20
11	5.90	2.64	6.28	4.94	4.88	3.26	3.64	3.93
12	4.74	3.52	6.52	4.93	7.30	5.94	3.70	5.65
24	22.8	13.7	14.3	16.9	8.96	5.68	6.30	6.98
36	20.6	11.8	11.6	14.7	6.88	6.48	4.44	5.93
48	24.2	6.64	6.90	12.6	2.63	3.46	3.32	3.14
60	14.2	2.94	3.46	6.88	2.50	1.22	2.86	2.19
72	1.92	2.51	1.73	2.05	2.75	1.03	1.39	1.72
84	1.75	3.09	1.97	2.27	2.74	1.33	2.39	2.15
96	1.03	3.13	1.21	1.79	2.87	0.70	1.62	1.73

Table S7. Total headspace H_2 (µmol) for *T. paralvinellae* at various time points when cells were grown in batch cultivation on brewery wastewater only and brewery wastewater plus formate

Time	Brewery wastewater only				Brewery wastewater plus formate			
(h)	1	2	3	Mean	1	2	3	Mean
2	-	3.94	9.46	6.70	149.41	140.46	-	144.93
3	4.10	4.62	12.39	7.04	212.21	244.50	229.53	228.75
4	9.45	7.71	14.07	10.41	257.46	378.37	385.98	340.60
5	14.01	13.72	-	13.87	354.20	527.43	628.61	503.42
6	19.08	19.50	34.31	24.30	402.51	657.54	919.99	659.99
7	23.75	29.85	40.76	31.45	537.28	806.41	879.48	741.06
8	28.08	38.78	76.29	47.71	655.61	772.47	256.47	561.52
9	33.54	40.60	93.83	55.99	700.98	300.85	48.37	350.07
10	38.26	67.99	116.44	74.23	440.89	56.26	46.98	181.38
11	47.15	85.40	132.98	88.51	66.71	51.11	58.10	58.55
12	54.99	104.98	144.5	101.49	69.71	62.58	60.25	64.18
24	71.18	127.82	150.16	116.39	35.09	31.25	31.30	32.55
36	43.36	63.66	89.21	65.41	22.02	21.14	23.66	22.27
48	22.10	22.66	32.90	25.89	6.68	10.70	13.67	10.35
60	7.75	3.61	8.88	6.75	1.53	4.84	5.86	4.08
72	2.24	-	-	0.75	0	1.75	2.26	1.34
84	-	-	-	-	-	-	0.57	0.19

Table S8. H_2 yield per cell (fmol/cell) for *T. paralvinellae* at various time points when cells were grown in batch cultivation on brewery wastewater only and brewery wastewater plus formate

Time	Brewery wastewater only				Brewery wastewater plus formate			
(h)	1	2	3	Mean	1	2	3	Mean
2	-	-	0.99	-	24.10	24.56	-	24.33
3	1.09	1.12	0.74	0.98	32.35	41.58	40.70	38.21
4	1.18	1.42	0.89	1.16	21.89	45.48	45.95	37.77
5	1.11	1.07	-	1.09	29.13	41.21	72.09	47.47
6	1.11	0.95	1.17	1.08	19.28	39.14	49.35	35.92
7	1.17	1.03	0.62	0.94	16.18	36.13	40.42	30.91
8	1.10	1.62	1.35	1.36	15.70	19.63	10.83	15.39
9	1.12	0.69	0.57	0.79	11.18	6.79	1.41	6.46
10	0.75	1.02	0.65	0.80	5.36	0.76	1.02	2.38
11	0.50	0.80	0.54	0.61	0.85	0.98	1.00	0.94
12	0.73	0.74	0.56	0.67	0.60	0.66	1.02	0.76
24	0.20	0.23	0.27	0.23	0.24	0.34	0.31	0.30
36	0.13	0.13	0.19	0.15	0.20	0.20	0.33	0.25
48	0.06	0.08	0.12	0.09	0.16	0.19	0.26	0.20
60	0.03	0.03	0.06	0.04	0.04	0.25	0.13	0.14
72	0.07	-	-	-	-	0.11	0.10	0.10
84	-	-	-	-	-	-	0.01	-

Table S9. Soluble chemical oxygen demand (mg COD/L) in spent growth media when *T. paralvinellae* was grown in batch cultivation on brewery wastewater only and brewery wastewater plus formate

Time	Brewery wastewater only				Brewery wastewater plus formate			
(h)	1	2	3	Mean	1	2	3	Mean
0	6161	6575	6481	6406	6940	6739	6833	6837
24	6199	6192	6250	6213	6437	6778	6370	6528
48	6070	6375	6402	6282	6572	6325	6350	6416
72	6160	6321	6185	6222	6351	6402	6279	6344
96	6223	6323	6534	6360	6370	6270	6217	6286

Table S10. Cell concentration, total headspace H_2, and H_2 yield per cell for *T. paralvinellae* when grown in a chemostat on brewery wastewater only (BW) and brewery wastewater plus formate (BW+F)

Time (h)	Cell concentration ($\times 10^7$ cells/ml)		Total headspace H_2 (μmol)		H_2 per cell (fmol/cell)	
	BW	BW+F	BW	BW+F	BW	BW+F
-4	0.52	1.39	5.68	172.97	0.69	7.78
-3	0.80	1.04	9.08	309.70	0.71	18.61
-2	1.11	1.96	16.58	630.87	0.93	20.11
-1	1.93	2.38	26.13	826.07	0.85	21.69
0	2.14	2.56	40.84	936.96	1.19	22.88
1	3.63	3.70	44.25	881.82	0.76	14.90
2	3.40	5.20	55.02	722.40	1.01	8.68
3	6.90	6.10	61.04	640.03	0.55	6.56
4	9.10	9.70	64.99	629.92	0.45	4.06
5	6.80	10.4	65.53	636.01	0.60	3.82
6	10.4	9.36	66.18	616.52	0.40	4.12
7	7.77	13.1	60.90	625.83	0.49	2.99
8	8.90	14.2	52.83	674.73	0.37	2.96
9	6.10	16.7	55.73	680.98	0.57	2.55
10	6.64	14.4	51.47	662.91	0.48	2.88
11	7.68	11.8	48.50	-	0.39	-
12	5.96	18.0	43.65	625.28	0.46	2.17
13	5.98	-	40.18	605.29	0.42	2.96
14	3.48	12.8	33.94	-	0.61	-
15	3.58	24.1	29.33	647.40	0.51	1.67
16		18.1		532.30		1.84
17		14.7		641.65		2.72
19		14.5		678.48		2.93
20		26.0		678.55		1.63
21		19.2		631.97		2.06
22		23.7		650.99		1.72
